# Desferal regulates hCtr1 and transferrin receptor expression through Sp1 and exhibits synergistic cytotoxicity with platinum drugs in oxaliplatin-resistant human cervical cancer cells *in vitro* and *in vivo*

**DOI:** 10.18632/oncotarget.10336

**Published:** 2016-06-30

**Authors:** Szu-Jung Chen, Ching-Chuan Kuo, Hsin-Yi Pan, Tsui-Chun Tsou, Szu-Ching Yeh, Jang-Yang Chang

**Affiliations:** ^1^ Institute of Clinical Pharmacy and Pharmaceutical Sciences, College of Medicine, National Cheng Kung University, Tainan, Taiwan, ROC; ^2^ National Institute of Cancer Research, National Health Research Institutes, Tainan, Taiwan, ROC; ^3^ Institute of Biotechnology and Pharmaceutical Research, National Health Research Institutes, Zhunan, Taiwan, ROC; ^4^ Division of Environmental Health and Occupational Medicine, National Health Research Institutes, Zhunan, Taiwan, ROC; ^5^ Division of Hematology/Oncology, Department of Internal Medicine, National Cheng Kung University Hospital, College of Medicine, National Cheng Kung University, Tainan, Taiwan, ROC

**Keywords:** oxaliplatin resistance, copper transporter hCtr1, transferrin transporter TfR1, desferal, specificity protein 1 Sp1

## Abstract

The development of resistance to platinum drugs in cancer cells severely reduces the efficacy of these drugs. Thus, the discovery of novel drugs or combined strategies to overcome drug resistance is imperative. In addition to our previous finding that combined D-penicillamine with platinum drugs exerts synergistic cytotoxicity, we recently identified a novel therapeutic strategy by combining an iron chelating agent desferal with platinum drugs to overcome platinum resistance in an oxaliplatin-resistant human cervical cancer cell line, S3. Further study demonstrated that the level of platinum–DNA adduct formation positively correlated with cell death in combination of desferal with platinums than that of each drug alone in S3 cells. Decrement of human copper transporter 1 (hCtr1) and transferrin receptor 1 (TfR1) expression involved in the development of platinum resistance in S3 cells. Moreover, desferal promoted the expression of hCtr1 through the upregulation of Sp1. The overexpression of Sp1 increased the expression of NF-κB and translocated it into the nucleus to bind to the TfR1 promoter region, which subsequently increased the expression of TfR1. Importantly, the cotreatment of oxaliplatin with desferal significantly potentiated the oxaliplatin-elicited antitumoral effect in the oxaliplatin-resistant xenograft animal model without any toxic effect observed. Taken together, these results demonstrated that the combination of desferal with oxaliplatin can overcome oxaliplatin resistance through the regulation of hCtr1 and TfR1, and may have beneficial effect for treatment of patient with oxaliplatin-refractory tumors.

## INTRODUCTION

The platinum-based drugs, included cisplatin, oxaliplatin, and carboplatin, were the most commonly used therapeutic agents for treating solid tumors in clinical [1]. However, accumulating evidences indicated that the development of drug resistance severely impedes and reduces the antitumor effect of these agents [2, 3].

Platinum-based drugs cause cell death through the formation of a covalent bond between the platinum moiety of a platinum-based drug and the purine base of DNA [4]. These intra- and inter-strand crosslinks of platinum–DNA complexes destroy the DNA structure, resulting in cell death [5]. Studies have reported that reduction in the intracellular accumulation of platinum–DNA adduct through the regulation of drug influx and efflux pumps, enhancement of DNA repair and drug detoxification, are major factors contributing to platinum resistance [5, 6].

Platinum drugs enter cells through diffusion or endocytosis. Studies have reported that human copper transporter 1 (hCtr1), a copper influx transporter, participates in the transportation of platinum drugs and is frequently downregulated in platinum-drug resistant variants [7, 8]. Two copper efflux transporters, copper-transporting ATPase 1 (ATP7A) and copper-transporting ATPase 2 (ATP7B), transport platinum drugs from the cytoplasm into subcellular compartments, primarily localized to the trans-Golgi network for subsequent efflux in a similar manner to their effect on copper [9]. This action reduces the cytotoxicity of platinum drugs and causes drug resistance. We and others have demonstrated that the decreased expression of hCtr1 and overexpression of ATP7A and ATP7B have a crucial role in the development of platinum resistance [10–14]. Moreover, our previous study reported that combination of D-penicillamine, a copper chelator, and platinum drugs exhibits synergistic interaction in oxaliplatin-resistant cancer cells through modulation of hCtr1 and ATP7A expression [13, 14]. Thus, we proposed that the metal chelators, such as iron chelators, may also be benefit in manipulation of copper transporters and sensitize resistant cancer cells with chemotherapeutic agents. In the present study, we first time demonstrated that combination of desferal, an iron-chelating agent, with platinum drugs synergistically inhibits tumor growth and promotes tumor cell death in oxaliplatin-resistant cells *in vitro* and in preclinical human xenograft animal model. The underlying mechanism for this synergism, at least in part, is through the upregulation of hCtr1 and transferrin receptor 1 (TfR1) by desferal, thereby subsequently increases the platinums-elicited cellular platinum–DNA adduct formation and cytotoxicity.

## RESULTS

### Combination effect of platinum drugs and desferal on SiHa and S3 cells

Oxaliplatin-resistant cells S3 were established from SiHa cells by exposure to increasing concentrations of oxaliplatin. The antiproliferative effect of platinum-based drugs and desferal in SiHa and S3 cells was illustrated in Table 1. As compared with parental SiHa cells, S3 cells were more resistant to oxaliplatin, followed by cisplatin and carboplatin, with the resistance index of approximately 77.6, 12.7, and 2.7, respectively. However, the IC_50_ value of desferal for both SiHa and S3 cells was almost similar.

**Table 1:**
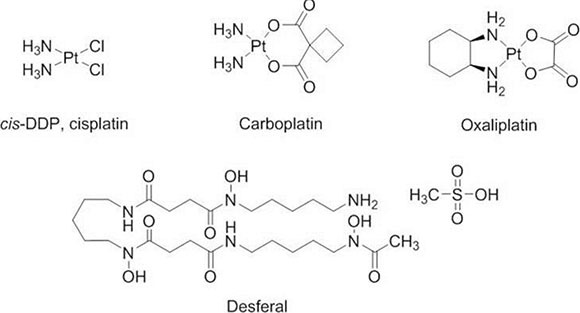
Sensitivity of parental SiHa cells and oxaliplatin-resistant S3 subline cells to platinum-based drugs and desferal

Drugs	SiHa	S3	Resistance Index (IC_50_ of S3/IC_50_ of SiHa)
	IC_50_(μM)	IC_50_(μM)	
Oxaliplatin	0.8 ± 0.1	62.1 ± 1.6	77.6
Cisplatin	1.2 ± 0.1	15.2 ± 1.5	12.7
Carboplatin	3.0 ± 0.8	8.1 ± 0.6	2.7
Desferal	74.7 ± 0.4	77.0 ± 1.6	-

Each value is presented as the means ± standard deviation (S.D.) of three independent experiments.

**Figure 1:**
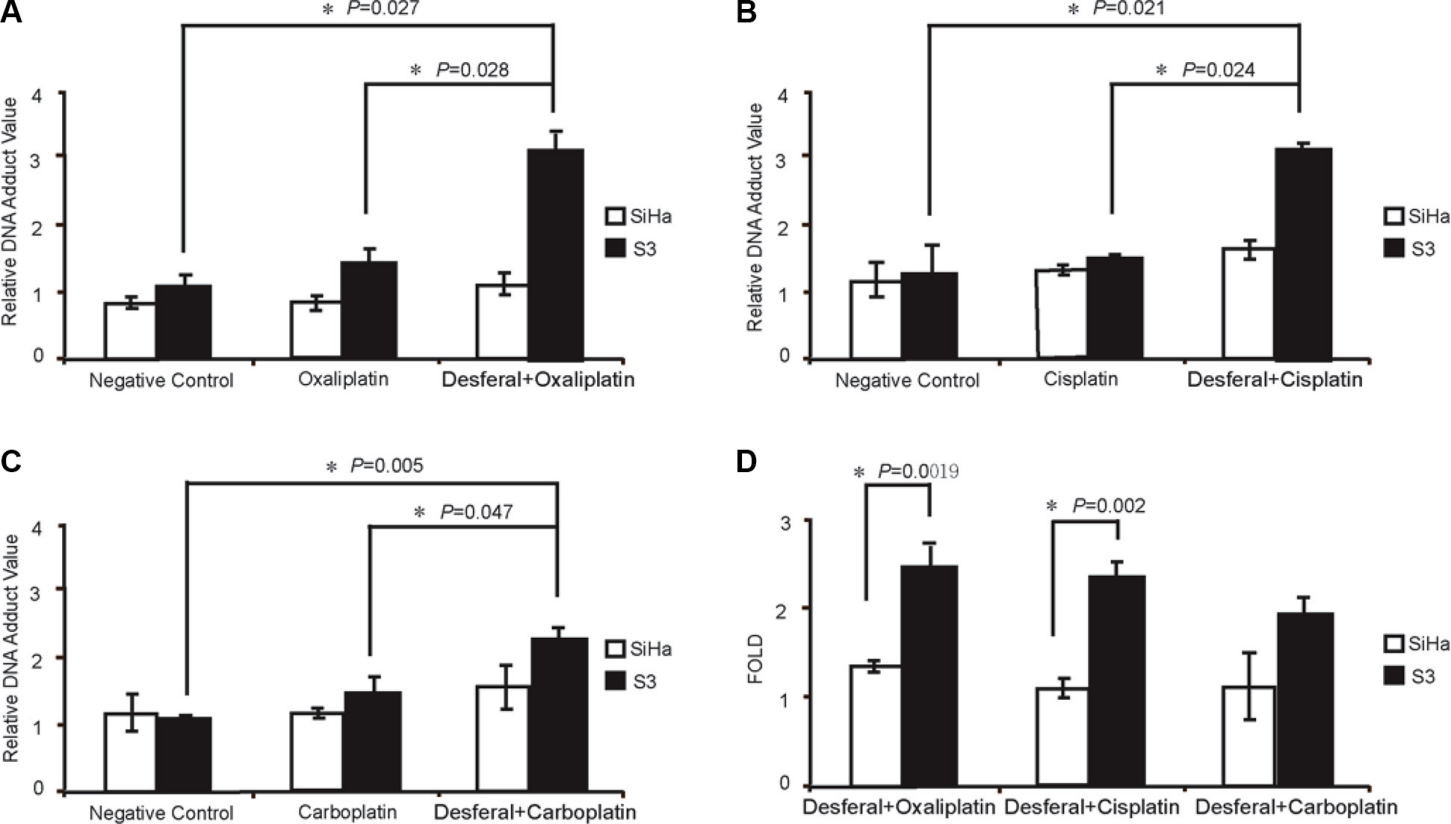
Desferal synergistically interacts with platinum drugs and increases platinum–DNA adduct formation in oxaliplatin-resistant S3 cells (**A**–**D**) Measurement of platinum–DNA adduct formation in cells treated with 1-fold of IC_50_ of platinum drugs (refer to Table 1) and desferal, as described in the Materials and Methods section. Each value is presented as the mean ± standard deviation (SD) of three independent experiments (*P* < 0.05).

To investigate the combination effect of desferal and platinum-based drugs, both SiHa and S3 cells were simultaneously treated with desferal and platinum-based drugs for 72 h. As listed in Table 2, the combination index (CI) of desferal with oxaplatin, cisplatin, and carboplatin was 0.51, 0.68, and 0.93 in S3 cells and 1.76, 1.18, and 1.37 in parental SiHa cells, respectively. We further determined whether the increased sensitivity of S3 cells toward platinums correlated to the increased the formation of intracellular platinum–DNA adduct. As the result, the formation of platinum–DNA adduct significantly increased in the combination regimens with desferal than platinum alone in S3 cells; however, this effect did not observe in parental cells (Figure 1A–1C). Compared with a single-drug treatment, the cotreatment of desferal with oxaliplatin or cisplatin significantly increased DNA adducts formation in S3 cells. However, no change in DNA adduct formation was observed in S3 cells treated either with carboplatin plus desferal or carboplatin alone (Figure 1D).

**Table 2:** Combination effect of desferal and platinum drugs, including oxaliplatin, cisplatin, and carboplatin, on SiHa and S3 cells

Drugs	SiHa	S3
	Combination Index (CI)	
Oxaliplatin + Desferal	1.76	0.51
Cisplatin + Desferal	1.18	0.68
Carboplatin + Desferal	1.37	0.93

The drug combination analysis was performed using the Chou and Talalay method. Graphs representing values of combination index (CI) versus fractional effect were automatically generated using CalcuSyn software. Effect is the fraction of cell death induced by drug treatment and ranges from 0 to 1, with 0 representing no cell death and 1 representing 100% cell death. The quantitative CI value defines addictive effect as CI = 1, synergism as CI < 1, and antagonism as CI > 1 in a combination of two drugs.

### Desferal increases the expression of hCtr1 and TfR1 through the upregulation of Sp1 in S3 cells

**Figure 2:**
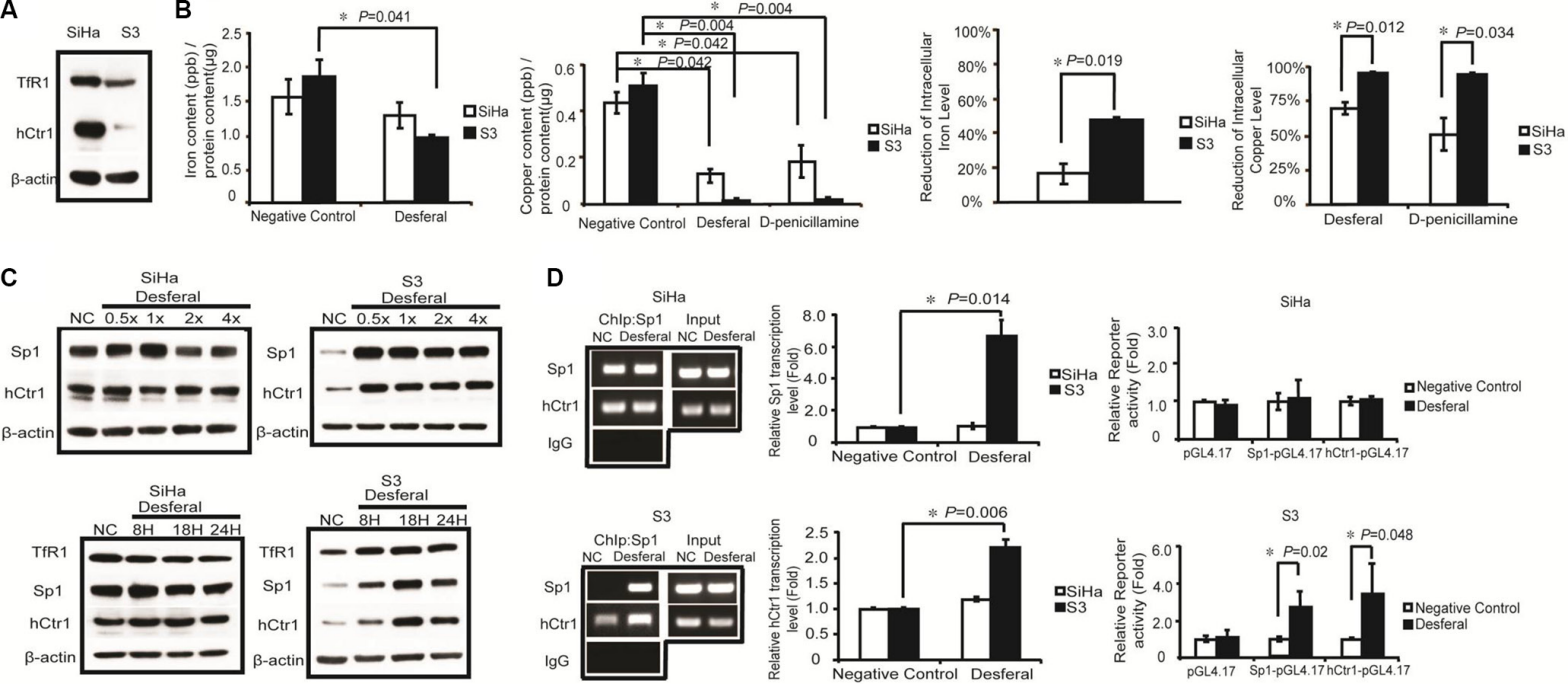
Effects of desferal on the concentration of intracellular iron and copper and expression of Sp1, hCtr1, and TfR1 (**A**) Western blot analysis of the expression levels of hCtr1 and TfR1 protein in SiHa and S3 cells. β-Actin served as an internal control. **(B)** SiHa and S3 cells were treated with 1-fold of IC_50_ of desferal or D-penicillamine. After 24 h, cells were lysed and subjected to mass spectrometry for determining intracellular iron and copper concentrations (*P* < 0.05). **(C)** SiHa and S3 cells were treated with folds of IC_50_ of desferal for 24 h. Cell lysates were then harvested and subjected to Western blot analysis. NC denotes negative control. SiHa and S3 cells were treated with 1-fold of IC_50_ of desferal for 8, 18, and 24 h. Cell lysates were then harvested and subjected to Western blot analysis. NC denotes negative control. **(D)** The effect of desferal on Sp1 binding to the Sp1 and hCtr1 promoter regions in both SiHa and S3 cells was analyzed through ChIP. The effect of desferal on the transcription levels of both Sp1 and hCtr1 genes was determined through RT-qPCR (*P* < 0.05). The effect of desferal on the promoter activity levels of both Sp1 and hCtr1 promoters was determined using the promoter assay (*P* < 0.05).

To determine the basal level of both hCtr1 and TfR1 proteins in SiHa and S3 cells, western blot analysis was performed. The results revealed that the expression level of hCtr1 and TfR1 was higher in SiHa cells than in S3 cells (Figure 2A). Furthermore, we examined the effect of desferal on intracellular iron and copper concentrations in both SiHa and S3 cells. As presented in Figure 2B, desferal treatment significantly reduced more intracellular iron and copper concentrations in S3 cells than in SiHa cells (48% vs.17%, *P* = 0.019; 96% vs.69%, *P* = 0.012). Previously, we demonstrated that the expression of hCtr1 is controlled by the transcription factor Sp1, which is negatively regulated by intracellular copper concentrations [14]. We further evaluated the effect of desferal on the expression of Sp1 and hCtr1 in both SiHa and S3 cells. We observed an increase in the expression of Sp1 and hCtr1 in the concentration- and time-dependent manner in S3 cells; however, desferal did not affect Sp1 and hCtr1 in SiHa cells (Figure 2C). Notably, desferal increased the expression of TfR1 in a time-dependent manner (Figure 2C). We further investigated whether the expression of hCtr1 is through the transcriptional regulation of Sp1 on the hCtr1 promoter in S3 cells. We observed that desferal treatment induced the binding of Sp1 to the hCtr1 promoter and increased the expression of hCtr1 (Figure 2D).

### Desferal treatment induces the expression of TfR1 through the Sp1–NF-κB p65-dependent pathway

**Figure 3:**
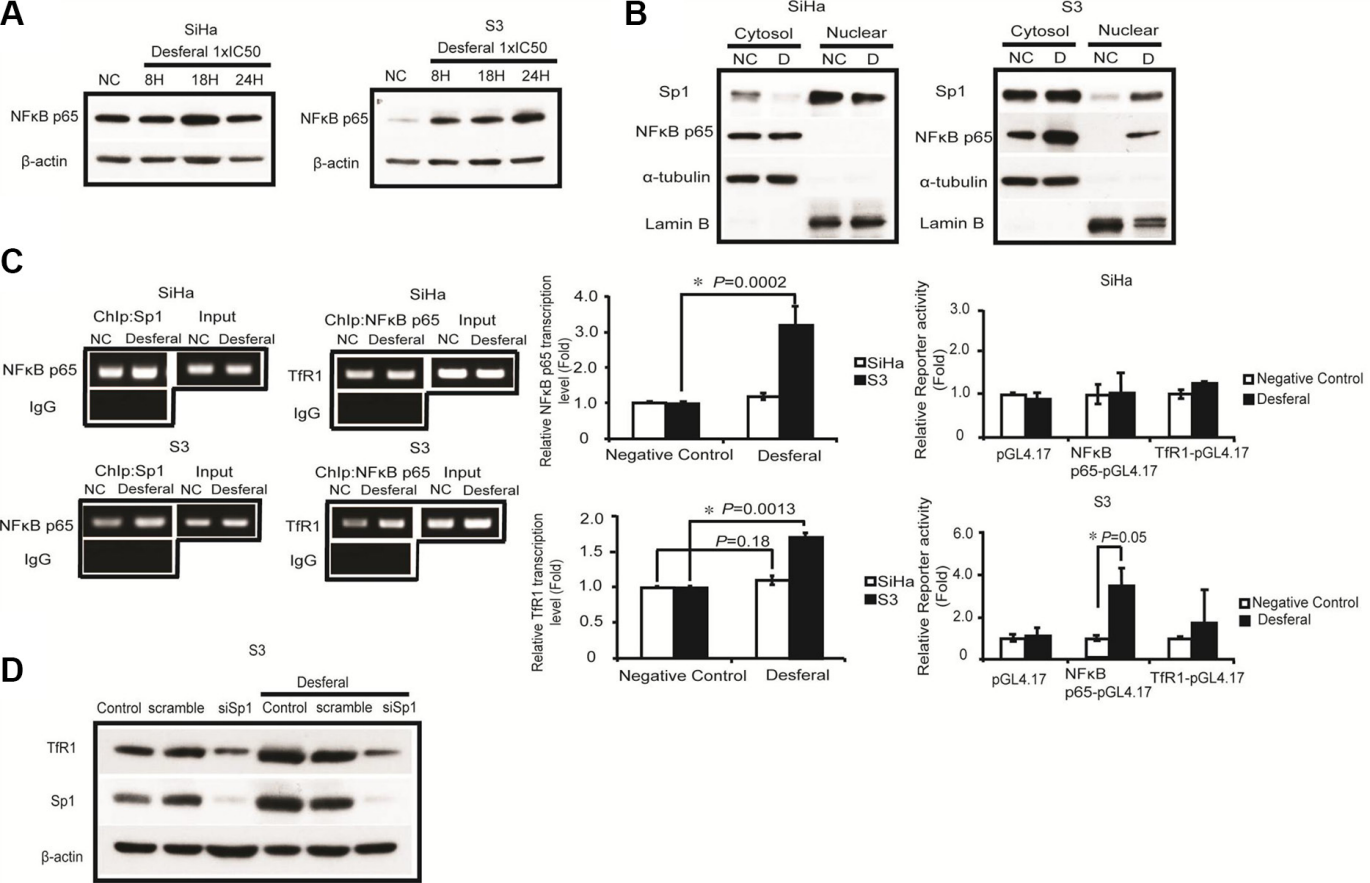
Desferal induced the expression level of TfR1 through the Sp1 -dependent pathway (**A**) Desferal induced NF-κB p65 expression in S3 cells in a time-dependent manner. Both SiHa and S3 cells were treated with desferal for 8, 18, and 24 h. Cells were then harvested and subjected to Western blot analysis. NC denotes negative control (**B**) The effect of desferal on the nuclear localization of Sp1 and NF-κB p65. Cells were treated with or without desferal for 24 h, and the cytosolic and nuclear fractions were then separated, as described in the Materials and Methods section. (**C**) Desferal induces the binding of Sp1 to the NF-κB p65 promoter region and that of NF-κB p65 to the TfR1 promoter region in S3 cells as demonstrated using a ChIP assay. Cells without desferal treatment served as the negative control (NC). Desferal significantly induced the transcription of NF-κB p65 and TfR1 in S3 cells as demonstrated using an RT-qPCR assay. The effect of desferal on the promoter activity levels of both NF-κB p65 and TfR1 promoters was determined using promoter assay (*P* < 0.05). (**D**) Desferal induced TfR1 expression through the Sp1-dependent pathway. S3 cells were transfected with either scramble control or Sp1-targeted siRNA, respectively, followed by treatment with desferal. Cells without transfection served as the control. β-actin was used as an internal control.

Several studies have reported that Sp1 regulates NF-κB p65 transcription [15, 16]. As presented in Figure 3A, our results revealed that desferal increased the expression of NF-κB p65 in S3 cells. Furthermore, we observed an increase in the nuclear expression of Sp1 and NF-κB p65 after treating S3 cells with desferal (Figure 3B). The nuclear accumulation of NF-κB p65 maybe related to its transcriptional activity after desferal treatment. Moreover, the activation of NF-κB p65 in LPS–IFNγ-treated macrophage cells was reported to be relative to the expression of TfR1 [17]. Therefore, we analyzed the TfR1 promoter sequence by using the QIAGEN EpiTect chromatin immunoprecipitation (ChIP) promoter binding site prediction and observed the presence of potential NF-κB-binding sites in the TfR1 promoter region. We hypothesized that the upregulation of TfR1 is through the regulation of NF-κB p65. We observed that NF-κB p65 promoter-bound Sp1 and TfR1 promoter-bound NF-κB p65 were increased in S3 cells treated with desferal (Figure 3C). However, this effect did not observe in SiHa cells. In addition, the transcription levels of TfR1 and NF-κB p65 significantly increased after desferal treatment in S3 cells (Figure 3C). To further determine whether Sp1 is responsible for the regulation of NF-κB p65 and TfR1 expression, we knocked down Sp1 expression by using a siRNA followed by treated cell with desferal. As the result, we found that the silence of Sp1 reduced the expression of TfR1 in S3 cells in both presence and absence of desferal conditions (Figure 3D).

### TfR1 serves as a transporter for platinum drugs

**Figure 4:**
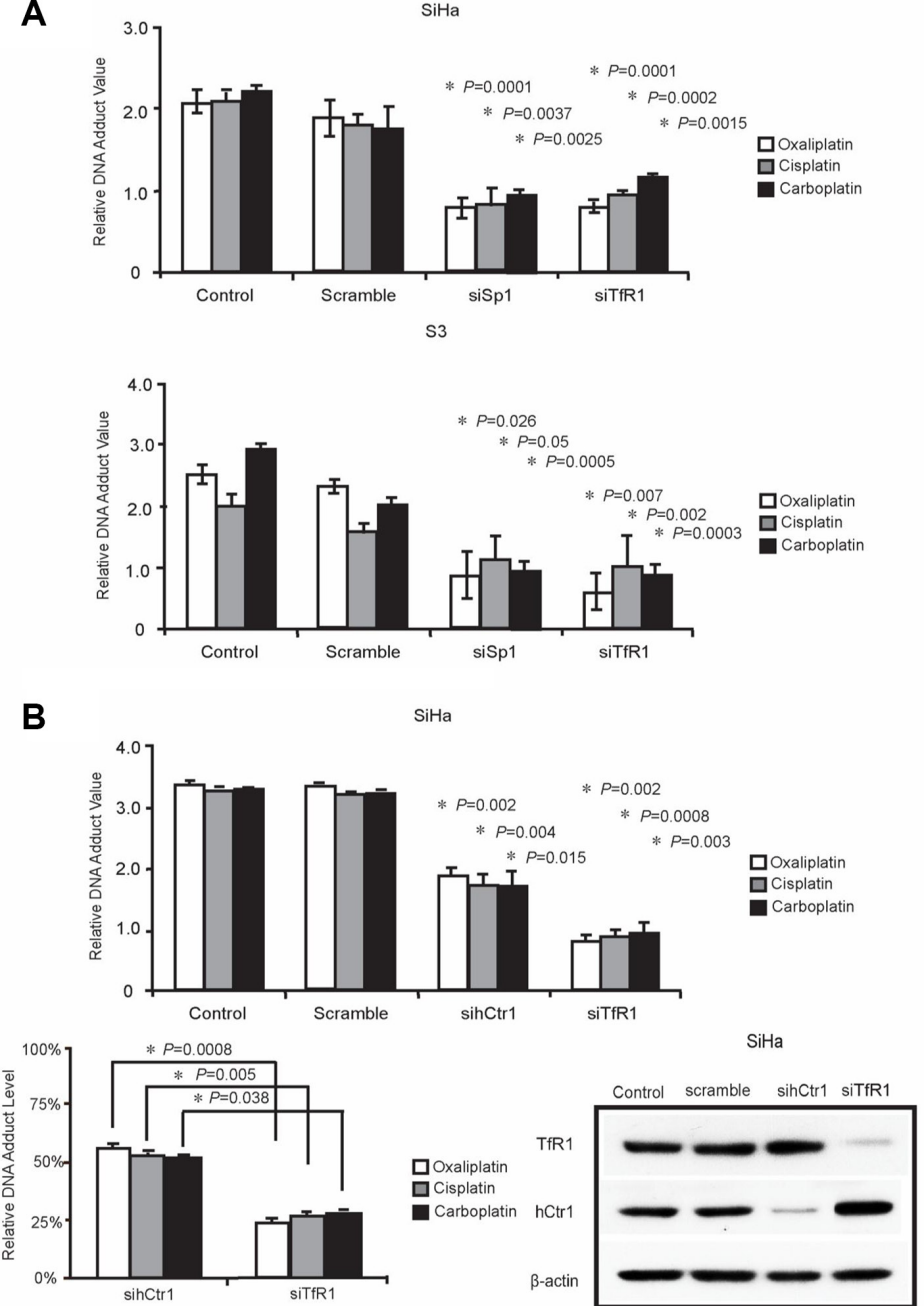
TfR1 promoted DNA adduct formation through Sp1 (**A**) Sp1 or TfR1-targeted were transfected into SiHa and S3 cells, respectively. 5-fold of IC_50_ platinum drugs including oxaliplatin, cisplatin, and carboplatin were incubated with cells for performing ELISA assay (*P* < 0.05), as described in Materials and Methods section. (**B**) SiHa cells were transfected with TfR1- or hCtr1-targeted siRNA for comparing the efficiency of transporting platinum drugs for performing ELISA assay (*P* < 0.05).

To investigate whether TfR1 is responsible for the transportation of platinum-based drugs, we knocked down the expression of TfR1 by using a siRNA in both SiHa and S3 cells, followed by treatment with platinum-based drugs, and subsequently measured platinum–DNA adduct formation. As presented in Figure 4A, platinum–DNA adduct formation significantly decreased in both SiHa and S3 cells after the downregulation of TfR1. We further compared DNA adduct formation after treatment with platinum-based drugs in hCtr1 or TfR1 knockdown cells. We observed that the downregulation of hCtr1 and TfR1 reduced platinum–DNA adduct formation (Figure 4B). Among them, silencing of TfR1 is more effective than knockdown of hCtr1 to reduce platinum–DNA adduct formation.

### Desferal increases the antitumor effect of oxaliplatin in an oxaliplatin-resistant tumor xenograft model

**Figure 5:**
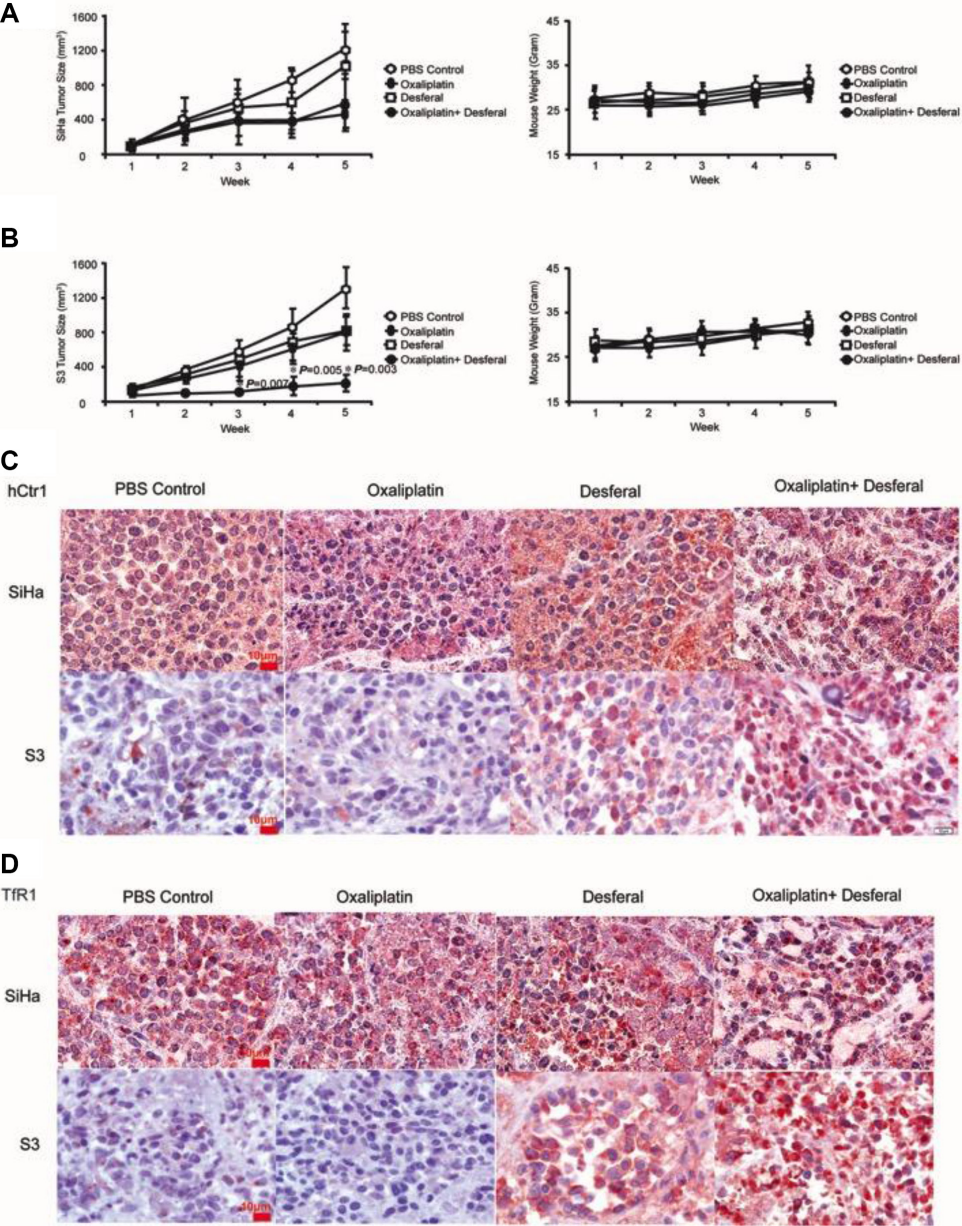
The antitumoral effect of oxaliplatin alone or in combination with desferal in the xenograft animal model Nude mice bearing SiHa cells **(A)** and S3 **(B)** cells were treated with PBS control (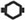), 5 mg/kg of oxaliplatin (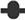), 200 mg/kg of desferal (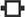), or a combination of oxaliplatin and desferal (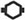) 3 times per week for 3 wk through a tail vein injection. Data are presented as the mean ± SD of tumor volume (mm^3^) of each time point (*n* = 5, *P < 0.05). Immunohistochemistry analysis of hCtr1 **(C)** and TfR1 **(D)** expression in tumor tissues (1000× magnification).

Our results support the notion that desferal increases the uptake of platinum-based drugs, which subsequently increases DNA adduct formation and sensitizes cells to death *in vitro*. We further examined the combination effect of desferal and oxaliplatin *in vivo*. Both SiHa and S3 tumor xenograft mouse models were used. Compared with vehicle controls, oxaliplatin delayed growth of both SiHa and S3 tumors. However, the antitumor effect increased only in S3 cells after the combination treatment of desferal with oxaliplatin (Figure 5B). In addition, we observed no significant body weight loss in these mice (Figure 5A–5B). We further assessed the expression levels of hCtr1 and TfR1 in tumor tissues through immunohistochemistry (IHC) staining. Consistent with our *in vitro* findings, desferal treatment considerably increased the expression of hCtr1 and TfR1 in S3 tumors (Figure 5C–5D). Taken together, our results demonstrate that the combination of desferal and oxaliplatin is an effective approach to inhibit the tumor growth in oxaliplatin-refractory tumors.

## DISCUSSION

In our previous study, we demonstrated that a copper-chelating agent could reverse oxaliplatin resistance through the regulation of the copper importer hCtr1 and exporter ATP7A [14]. We further observed that the expression level of TfR1 was lower in S3 cells than in parental cells (Figure 2A). These findings prompted us to identify iron-chelating agent and investigate the combination effect of iron chelating agents with platinum drugs in oxalipaltin resistant tumor models. Indeed, we noted that desferal, an iron-chelating agent, exhibited synergistic interaction with oxaliplatin or cisplatin to inhibit tumor cell growth in oxaliplatin-refractory tumors.

Iron is an essential element for cell replication, metabolism, and growth. When iron is insufficient, cells increase TfR1 expression to import more iron into the cell. Desferal, an iron chelator, is currently used to treat iron-overload diseases [18, 19]. The potential of desferal and its analogs in cancer therapy has emerged from the finding that cancer cells require higher iron concentrations during DNA synthesis and growth than normal cells do [20]. Studies have reported that desferal removes iron from cancer cells and prevents iron uptake by TfR1, thus exerting the antiproliferative effect [21, 22]. In addition to its iron-chelating effect, desferal can eliminate free copper. Van Reyke *et al.* reported that under optimal conditions, one mole of desferal is sufficient to reduce three moles of copper [23]. In an animal study, another iron chelating drug, deferasirox, increased copper levels by 2-fold in the kidney compared with those in the tumor. The copper–deferasirox complex was trapped in the kidney during the filtration, resulting in a significant increase in copper levels in the kidney [21, 22]. These findings suggest that these compounds exhibit multi-chelating activity, which broadens their antitumor effect alone or in combination with other anticancer agents. Consistent with these findings, our data further confirmed that desferal has multi-chelating activity (Figure 2B), which synergizes with the effect of platinum-based drugs to treat cancer.

Platinum drugs mainly enter cells through passive diffusion or endocytosis. The net intracellular level of platinum drugs is balanced between influx and efflux. Studies have reported that several transporters have different functions to regulate the net intracellular level of platinum drugs [24, 25]. Evidence has indicated that copper transporters have a crucial role in transporting platinum drugs into cells [8, 26]. The major copper influx transporter hCtr1 has been reported to assist the uptake of CDDP, carboplatin, and oxaliplatin and to regulate their cytotoxicity in yeast and mammalian cells [27, 28]. Furthermore, two copper efflux transporters, ATP7A and ATP7B, transport platinum drugs from the cytoplasm into subcellular compartments, primarily localized to the trans-Golgi network for subsequent efflux in a similar manner to their effect on copper [9]. This action reduces the cytotoxicity of platinum drugs and causes drug resistance. We have previously demonstrated that the downregulation of hCtr1 and upregulation of ATP7A render cells resistant to platinum drugs [29]. In addition to the alteration of copper transporters, we observed that the expression level of TfR1 was lower in S3 cells than in parental cells (Figure 2A). These results prompted us to investigate whether TfR1 has a role in transporting platinum drugs. The downregulation of TfR1 by using a siRNA reduced the formation of intracellular platinum–DNA adduct (Figure 4A). Moreover, the reduction in DNA adduct formation was significantly higher in cells treated with siTfR1 than in cells treated with sihCtr1. This is the first study to report that TfR1 can transport platinum drugs into cells, probably with a higher efficiency than that of hCtr1.

Consistent with these findings, we observed that desferal reduced intracellular copper concentrations and increased Sp1, hCtr1, and TfR1 expression levels both in a dose- and time-dependent manner in S3 cells (Figure 2C). Moreover, the ChIP assay revealed that Sp1 increased the binding of Sp1 to hCtr1 promoter regions and then increased Sp1 and hCtr1 transcription and translation in S3 cells treated with desferal (Figure 2D). Cumulative evidence has indicated that Sp1 directly regulates NF-κB p65 transcription [15, 16]. By using QIAGEN EpiTectChIP promoter binding site prediction, we observed two putative NF-κB p65 binding sites on the TfR1 promoter. We then hypothesized that desferal affects TfR1 expression by increasing the binding of Sp1 to NF-κB p65 binding sites, which subsequently increases NF-κB p65 expression and binding to the TfR1 promoter region. Our results revealed that desferal increased NF-κB p65 expression in S3 cells (Figure 3A). In addition, desferal treatment increased the nuclear accumulation of Sp1 and NF-κB p65 only in S3 cells (Figure 3B). Taken together, these data confirmed for the first time that an iron chelator could increase TfR1 expression through the Sp1–NF-κB–TfR1 axis (Figure 3C and 3D). Compared the negative control from nuclear extraction assay (Figure 3B) and ChIP assay (Figure 3C), Sp1 in S3 cells presented the lower expression than in SiHa cells. Therefore, lower transcription of Sp1 in S3 cells caused the low expression of TfR1. Given the previous finding that transferrin receptor promoter contains several Sp1-like binding sites [30], in this study we revealed that TfR1 expression was through the Sp1-NF-kB-TfR1 axis. Therefore, Sp1 may also directly bind transferrin receptor promoter and collaborate with NF-kB in upregulating transferrin receptor expression.

We assessed the antitumor activity of oxaliplatin in combination with or without desferal in human tumor xenografts in mice. Compared with SiHa tumors, the combination of oxaliplatin and desferal significantly increased the antitumor activity of oxaliplatin in S3 tumors (Figure 5A–5B). Furthermore, IHC analysis revealed that the expression levels of hCtr1 and TfR1 increased only in S3 tumor tissues treated with desferal (Figure 5C–5D). These results suggest that desferal restores the sensitivity of cancer cells to platinum-based drugs through the upregulation of hCtr1 and TfR1. The transferrin receptor is responsible for major iron homeostasis and supports growth of cancer cells [31, 32]. Because malignant cells have increased transferrin receptor expression, this receptor is widely considered as an accessible portal for drug delivery into cancer cells and is becoming a potential target for cancer therapy [33–35]. A phase 1 clinical study reported a partial response of 87% after using cisplatin-conjugated transferrin to treat patients with advanced cancers [36]. In addition, Fu *et al.* reported that in patients with ovarian cancer refractory to platinum drug treatment, platinum resistance was overcome through the use of a copper-lowering agent [37]. In addition to a copper chelator, our data provide evidence that an iron chelator can overcome platinum resistance through the regulation of hCtr1 and TfR1.

In conclusion, we demonstrated that the synergistic killing effect of the combination of desferal and oxaliplatin on oxaliplatin-resistant cells is through the modulation of the expression of a copper transporter and transferrin receptor *in vitro* and *in vivo*. In addition, we reported that the upregulation of transferrin receptor is through the Sp1–NF-κB-dependent pathway. These findings provide a potential therapeutic strategy for patients having oxaliplatin resistance and decreased hCtr1 and TfR1 expression.

## MATERIALS AND METHODS

### Reagents

Platinum-based compounds, oxaliplatin, cisplatin, and carboplatin, were obtained from Sanofi (NY, USA, 4F121A), Fresenius Kabi (India, 871QZ01001), and Pharmachemie B.V. (Haarlem, Netherlands, 12D7MD), respectively. D-penicillamine (P4875–1G) and desferal (D9533–1G) were purchased from Sigma-Aldrich. Primary antibodies, hCtr1 (sc-66847) and NF-κB p65 (sc-109), were purchased from Santa Cruz (CA, USA); Sp1 (07–645), α-tubulin (2508783), and TfR1 (ab84036) were purchased from Millipore Corporation (MA, USA) and Abcam (MA, USA), respectively. Horseradish peroxidase (HRP)-conjugated secondary antibodies (sc-2055, sc-2054) were purchased from Santa Cruz (CA, USA).

### Cell lines and culture

SiHa parental cells were from ATCC and cultured in DMEM medium (Thermo Scientific, Utah, USA, SH30003.02) supplemented with 5% fetal bovine serum (Thermo Scientific, Utah, USA, SH30071.03), 100U/mL of penicillin, and 100μg/mL of streptomycin–glutamate (Thermo Scientific, Utah, USA, 10378016) in a 5% CO_2_ incubator (humidified atmosphere) at 37°C. S3 cells, an oxaliplatin-resistant subclone of SiHa cells, were cultured in DMEM medium supplemented with 10% fetal bovine serum, 100 U/mL of penicillin, 100 μg/mL of streptomycin–glutamate, and 2 μg/mL of oxaliplatin. For the following assays, resistant cells were cultured in the medium without oxaliplatin to prevent the interference caused by oxaliplatin.

### Cell growth inhibition assay and combination index

For the cell growth inhibition assay, both SiHa and S3 cell lines were subjected to desferal, oxaliplatin, cisplatin, or carboplatin treatment, respectively, for 72 h. At the end of the treatment, cell growth was measured using a crystal violet stain assay. Cell growth inhibition determined by the dose that caused 50% cell death was designated as IC_50_. Resistance index was calculated using the following equation: *(IC*_50_ *of S3 cells)/(IC*_50_ *of SiHa cells)*. The CI between desferal and platinum drugs, including oxaliplatin, cisplatin, and carboplatin, was obtained using the Calcusyn software program, Version 1 (Biosoft, Cambridge, UK), which is based on the median effect equation of Chou and Talalay [38, 39]. The analysis used in this study was under the assumption of mutual no exclusiveness of the mechanism of drug action. On the basis of the theory of Chou and Talalay, CI defines additive effect as CI = 1, synergism as CI < 1, and antagonism as CI > 1 in a combination of two drugs.

### DNA adduct measurement

SiHa and S3 cells were treated with a 1-fold IC_50_ dose of oxaliplatin, carboplatin, and cisplatin either alone or in combination with desferal for 6 h. DNA was extracted and processed according to manufacturer instructions (Promega, Madison, USA, A1125). DNA adducts were measured using ELISA; each sample was coated on a 96-well ELISA plate (Sigma-Aldrich, CLS2525–10EA) and probed with specific monoclonal ICR4 antibodies (Millipore, MA, USA, Q2296671) to intrastrand adducts. For detection, 1-Step Ultra TMBELISA (Thermo Scientific, Utah, USA, PE1814394) was added to each sample and the reaction was stopped by adding 1 M sulfuric acid. The absorption wavelength was measured at 450 nm by using a microplate reader (Spectramax M5 plate reader, Molecular Devices) [40].

### Measurement of intracellular iron and copper concentrations

SiHa and S3 cell lysates were harvested using a lysis buffer (0.5% NP-40 and 10% glycerol in phosphate-buffered saline) before desferal or D-penicillamine treatment for 24 h or without treatment as a control. The protein concentration of whole-cell lysates was quantified and iron and copper concentrations were measured using Perkin Elmer SCIEX ELAN 6100 ICP mass spectrometer (Shelton, CT, USA).

### Small interference RNA transfection

Sp1-, NF-κB p65-, and TfR1-specific human small interfering RNAs (siRNAs) were purchased from Invitrogen (Waltham, USA, s13318, s11914, and s727). siRNA oligonucleotides were introduced into cells by using the oligofectamine transfection reagent according to manufacturer instructions.

### Western blot analysis

Cells were initially seeded at a density of 1 × 10^6^ in 100-mm^2^ dishes. After treatment, cells were lysed using a cell lysis buffer (Sigma-Aldrich, C2978) with 1 mM DTT, 1 mM PMSF, and a protease inhibitor (Roche, Mannheim, Deutschland, 04693116001) and vortexed for 3 seconds then incubated on ice for 1 h. Lysates were centrifuged at 4°C at 13,200rpm for 15 minutes. After centrifuging, supernatant was transferred to the new eppendorf. Further, 100 μg of cell lysates were separated using 10% SDS-PAGE at 100 v and were then transferred on polyvinylidinedifluoride membranes. The target proteins were probed with specific primary antibodies and HRP-conjugated secondary antibodies. The immunoreactive bands were detected using the ECL method and visualized on Kodak Bio-MAX MR film.

### Chromatin immunoprecipitation PCR

SiHa and S3 cells were treated with or without desferal for 24 h and then were crosslinked with 37% formaldehyde for 10 min at 37°C. The cells were then harvested and processed for DNA fragmentation through sonication on ice. The resultant chromatin–DNA fractions were purified using a ChIP assay kit (Millipore, 2487694) according to manufacturer instructions. Chromatin was immunoprecipitated at 4°C by using anti-Sp1, anti-NF-κB p65, and normal IgG (negative control). Primer sequences used were as follows: Sp1 on Sp1 promoter: forward 5′-CTTGGAGAGCAAGCGAGTCT-3′, reverse 5′-TGGACTCATCCTTACCGCTC-3′; Sp1 on hCtr1 promoter: forward 5′-CGGTCTCTGGACCGA AAGTA-3′, reverse 5′-CTCCGAATCTTAACCCGTCA-3′; Sp1 on NF-κB p65 promoter: forward 5′-AACTAATA ATAGGCCGGGCG-3′, reverse 5′-CGTGTTAGCCAGG ATGGTTT-3′; and NF-κB p65 on TfR1 promoter: forward 5′-CTGCTTCTTGAGGGAAAACG-3′, reverse 5′-TTAGG GGAGAACGCATCTGA-3′.

### Quantitative reverse transcription PCR

Total RNA was extracted from SiHa and S3 cell lines by using TRIzol® reagent (Thermo Fisher, 74108). First-strand cDNA was synthesized using the Super Script III first strand synthesis system according to manufacturer instructions. The transcription level of Sp1, hCtr1, NF-κB p65, and TfR1 was quantified using the Applied Biosystems 7500 Real-Time PCR system, with RPL13A as an internal control. Primer sequences used were as follows: hCtr1: forward 5′-AAGGGTGGTGGAATAATTGCAG-3′, reverse 5′-CTCACGAACTTGTGCCAGTAG-3′; Sp1: forward 5′-AGTTCCAGACCGTTGATGGG-3′, reverse 5′-GTTTGCACCTGGTATGATCTGT-3′; NFκB p65: forward 5′- GCAGAGGGGAATGCGTTTTAG-3′, reverse 5′-AGAAGGGTATGTTCGGTTGTTG-3′; and TfR1: forward 5′-ACCATTGTCATATACCCGGTTCA-3′, reverse 5′-CAATAGCCCAAGTAGCCAATCAT-3′.

### Gene reporter assay

The reporter gene plasmids of Sp1-, hCtr1-, TfR1-, and NF-κB p65-pGL4.17 were purchased from GENEWIZ (product numbers: B24656–1/V125740, B24656/V125742, B26543–1/M76364, and B26543–2/M76368). In brief, plasmids were introduced into cells by using the oligofectamine transfection reagent for 24 h and then processed using the Dual-Luciferase 1000 Assay system (Promega, Madison, USA, E1910) according to manufacturer protocol.

### Nuclear extraction assay

Samples were lysed using 500μL of buffer A (10 mM HEPES, 1.5 mM MgCl_2_, 10 mM KCl, 0.5 mM DTT, and 0.05% NP-40; pH 7.9). After centrifugation at 3,000 rpm for 10min at 4°C, the supernatant was collected as a cytosolic fraction. The pellet was washed three times with a wash buffer (10 mM HEPES, 1.5 mM MgCl_2_, and 10 mM KCl; pH 7.9) and then lysed using 450 μL of buffer B (5 mM HEPES, 1.5 mM MgCl_2_, 0.2 mM EDTA, 0.5 mM DTT, 26% glycerol, and 300 mM NaCl; pH 7.9). The supernatant was collected after centrifugation at 13,200 rpm for 20min at 4°C as a nuclear fraction. 100 μg of cytosolic and nuclear fractions were subjected to gel electrophoresis and analyzed through immunoblotting.

### Animal experiments and immunohistochemistry study

All the animal experiments were conducted according to the protocols of the Institutional Animal Care and Use Committee (American Association for Laboratory Animal Science). The male athymic (NCR nu/nu) nude mice (aged 3–4 wk) were housed in a pathogen-free environment. Animals were subcutaneously inoculated on the dorsal lumbosacral region with 1 × 10^7^ SiHa cells or 2 × 10^7^ S3 cells in a total volume of 100 μL with a growth-factor-reduced basement membrane. When the tumor volume reached 70–100 mm^3^, the animals were randomly divided into four groups, with 5 animals in each group. The mice were treated with indicated chemicals and drugs through a tail vein injection three times a week for subsequent 3 weeks. During the treatment, tumor volume and weight were evaluated three times per week. The tumor masses were removed on the fifth week of the study and were subjected to IHC staining procedures.

### Statistical analysis

Student's *t*-test was used to analyze the quantitative and statistical results. All data were carried out in triplicate and expressed as a mean and standard deviation (SD). A *P* value of < 0.05 was considered statistically significant.
